# Common Variable Immunodeficiency Associated With Connective Tissue Diseases: A Report of Two Cases

**DOI:** 10.7759/cureus.111910

**Published:** 2026-07-01

**Authors:** Loudiyi Sara, Soukaina Smaini, Karima Marc

**Affiliations:** 1 Pulmonology Department, Moulay Youssef Hospital, Centre Hospitalo-Universitaire (CHU) Ibn Sina, Rabat, MAR

**Keywords:** autoimmunity, common variable immunodeficiency, connective tissue diseases, hypogammaglobulinemia, interstitial lung disease

## Abstract

Common variable immunodeficiency (CVID) is the most frequent symptomatic primary immunodeficiency in adults and is increasingly recognized as a disorder of immune dysregulation. Autoimmune diseases, including connective tissue diseases, may precede the diagnosis of CVID and contribute significantly to morbidity, particularly through pulmonary involvement. We report two adult women patients with connective tissue diseases (rheumatoid arthritis and primary Sjögren’s syndrome) complicated by recurrent respiratory infections, interstitial lung disease, and severe hypogammaglobulinemia. In both cases, the diagnosis of CVID was established after a prolonged diagnostic delay, and immunoglobulin replacement therapy was indicated. These cases highlight the importance of considering CVID in patients with connective tissue diseases who present with recurrent infections or unexplained pulmonary involvement. Early diagnosis and timely initiation of immunoglobulin replacement therapy are essential for improving outcomes and preventing irreversible complications.

## Introduction

Common variable immunodeficiency (CVID) is the most prevalent symptomatic primary immunodeficiency in adults [1]. It is defined by hypogammaglobulinemia, impaired antibody responses, and exclusion of secondary causes of immunodeficiency [2]. Although recurrent sinopulmonary infections are the classical presentation, non-infectious complications, including autoimmunity and granulomatous disease, are now recognized as major contributors to morbidity and mortality [3].

Autoimmune manifestations may precede the diagnosis of CVID by several years [4,5]. While autoimmune cytopenias are the most common, connective tissue diseases such as Sjögren’s syndrome and rheumatoid arthritis have also been reported [6,7], reflecting underlying immune dysregulation [8,9].

Pulmonary involvement is a frequent and severe complication of CVID. Beyond recurrent infections and bronchiectasis, inflammatory and interstitial lung diseases--particularly granulomatous lymphocytic interstitial lung disease (GLILD)--are commonly associated with autoimmune phenotypes [10,11]. Delayed recognition of CVID in patients initially treated for connective tissue diseases may result in inappropriate immunosuppression and progressive lung damage [12].

In this report, we describe two cases of CVID associated with connective tissue diseases, namely rheumatoid arthritis and primary Sjögren’s syndrome, both complicated by recurrent respiratory infections and pulmonary involvement. These cases highlight the diagnostic challenges posed by overlapping autoimmune and immunodeficiency features and emphasize the importance of early immunological evaluation to prevent irreversible pulmonary complications.

## Case presentation

Case 1

A 52-year-old woman with rheumatoid arthritis treated with long-term low-dose oral corticosteroids and methotrexate presented with a two-year history of recurrent respiratory tract infections and progressive exertional dyspnea.

High-resolution computed tomography (HRCT) of the chest revealed bilateral nodules and micronodules, later evolving toward features of infectious pneumonitis with retractile bronchiectasis (Figure 1). Laboratory investigations and pulmonary function test findings are summarized in Table 1.

Microbiological investigations, including GeneXpert testing for *Mycobacterium tuberculosis* and cytobacteriological examination of sputum, were negative. HIV serology was negative.

Based on the history of recurrent infections, radiological findings, and immunological abnormalities, a diagnosis of CVID was established, and immunoglobulin replacement therapy (IgRT) was initiated.

**Figure 1:**
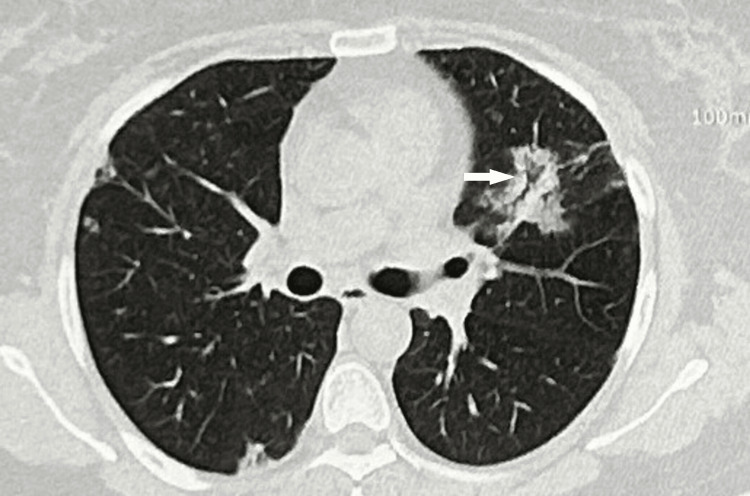
Axial chest CT scan showing bilateral nodules and micronodules, with subsequent evolution to features of infectious pneumonitis and traction bronchiectasis (white arrow).

**Table 1:** Laboratory and pulmonary function test findings in the two patients. FVC: forced vital capacity; FEV1: forced expiratory volume in 1 second; TLC: total lung capacity; Ig: immunoglobulin; HIV: human immunodeficiency virus. *Reference values for gamma globulins may vary according to laboratory standards.

Parameter	Case 1	Case 2	Reference range
Age (years)	52	49	-
Underlying connective tissue disease	Rheumatoid arthritis	Primary Sjögren’s syndrome	-
Lymphocyte count	Normal	900/µL	1000–4000/µL
Serum protein electrophoresis	Hypogammaglobulinemia: 1.5 g/L	Hypogammaglobulinemia: 0.9 g/L	6–8 g/dL gamma globulins*
Immunoglobulin G (IgG)	<0.3 g/L	1.2 g/L	7-16 g/L
Immunoglobulin A (IgA)	<0.05 g/L	3.01 g/L	0.7-4 g/L
Immunoglobulin M (IgM)	5.57 g/L	0.15 g/L	0.4-2.3 g/L
IgG subclasses	Decreased IgG2, IgG3, IgG4	Not available	-
Forced vital capacity (FVC)	2.15 L (83%)	1.71 L (53%)	>80% predicted
Forced expiratory volume in 1 second/forced vital capacity (FEV1/FVC ratio)	93%	82%	>70%
Total lung capacity (TLC)	92%	68%	80-120% predicted
24-hour urinary protein excretion	Normal	Normal	<150 mg/24 h
GeneXpert for *Mycobacterium tuberculosis*	Negative	Negative	Negative
Sputum cytobacteriological examination	Negative	Negative	Negative
HIV serology	Negative	Negative	Negative

Case 2

A 49-year-old woman had been followed since 2016 for primary Sjögren’s syndrome. Her history was notable for recurrent pulmonary embolism and bronchiectasis with chronic Pseudomonas aeruginosa colonization. She developed progressive dyspnea, sicca symptoms, and diffuse arthralgia.

HRCT of the chest showed bilateral bronchiectasis with centrilobular micronodules and interstitial changes (Figure 2). Laboratory investigations and pulmonary function test findings are summarized in Table 1.

Microbiological investigations, including GeneXpert and cytobacteriological examination of sputum, were negative. HIV serology was negative.

The combination of connective tissue disease, recurrent infections, interstitial lung disease, hypogammaglobulinemia, and restrictive ventilatory defect led to the diagnosis of CVID, and immunoglobulin replacement therapy was initiated.

**Figure 2:**
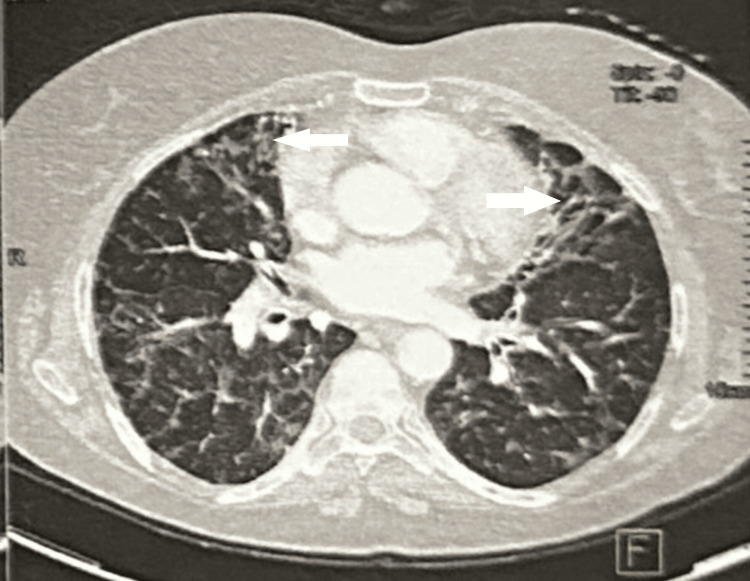
Axial chest CT scan showing bilateral bronchiectasis with centrilobular micronodules and interstitial changes (white arrows).

## Discussion

These two cases illustrate the complex and often underrecognized association between CVID and connective tissue diseases. Autoimmune manifestations occur in approximately 20-40% of patients with CVID and are increasingly considered a core feature rather than a coincidental comorbidity [4,13]. Patients with autoimmune phenotypes experience a longer diagnostic delay compared with those presenting with recurrent infections alone, frequently exceeding six years [5,6].

The pathophysiological basis of autoimmunity in CVID reflects profound immune dysregulation [8,9,14]. Defects in B-cell maturation, impaired generation of class-switched memory B cells, expansion of autoreactive B-cell clones, and abnormalities in regulatory T-cell function contribute to the breakdown of immune tolerance. Chronic antigenic stimulation from recurrent or persistent infections may further amplify autoimmune phenomena through sustained inflammatory signaling and molecular mimicry [15].

Among connective tissue diseases, Sjögren’s syndrome is the most frequently reported in association with CVID [6,7]. Sicca symptoms and lymphocytic sialadenitis may precede the diagnosis of immunodeficiency, as observed in our second patient. Hypogammaglobulinemia is atypical in primary Sjögren’s syndrome and should prompt reconsideration of the diagnosis. Failure to recognize underlying CVID may lead to inappropriate immunosuppressive treatment, increasing infectious risk and accelerating organ damage [3,4]

Rheumatoid arthritis, illustrated by our first case, is also well described in CVID, although its true prevalence is likely underestimated [6]. Long-term corticosteroid or immunosuppressive therapy may mask recurrent infections and delay recognition of immunodeficiency. These observations emphasize the need for systematic immunoglobulin measurement in patients with connective tissue diseases who present with unusual infectious patterns or poor treatment tolerance [12].

Pulmonary involvement represents a central determinant of morbidity and mortality in CVID, particularly in patients with autoimmune features [10,11,16]. Bronchiectasis, present in both of our cases, results from recurrent infections and chronic immune-mediated inflammation. Inflammatory lung diseases, such as granulomatous lymphocytic interstitial lung disease (GLILD), occur preferentially in patients with autoimmune and lymphoproliferative phenotypes and are associated with reduced survival. Early identification of CVID is therefore crucial to prevent irreversible pulmonary damage [10,11].

Immunoglobulin replacement therapy (IgRT) remains the cornerstone of CVID management and significantly reduces the frequency and severity of infections while improving survival [17]. Beyond its anti-infective role, IgRT has immunomodulatory properties that may stabilize certain autoimmune manifestations. Nevertheless, some patients require additional immunosuppressive or targeted therapies, which should be carefully balanced against infection risk and ideally managed in a multidisciplinary setting [3,17].

Both cases demonstrate the coexistence of connective tissue diseases with CVID, highlighting distinct clinical patterns. The first patient, with rheumatoid arthritis, presented with preserved pulmonary function and severe IgG subclass deficiencies, while the second patient, with Sjögren’s syndrome, exhibited restrictive lung disease and lymphopenia. These differences illustrate the heterogeneity of CVID presentations and the variable impact of autoimmune comorbidities on organ involvement. Both cases reinforce the need for early immunological evaluation in patients with connective tissue diseases and atypical infectious patterns, consistent with literature emphasizing delayed diagnosis, pulmonary complications, and the crucial role of IgRT in improving outcomes [1-17].

## Conclusions

CVID is a complex immune disorder frequently associated with autoimmune diseases, including connective tissue diseases such as Sjögren’s syndrome and rheumatoid arthritis. Autoimmune manifestations may precede the diagnosis of immunodeficiency and are associated with significant diagnostic delay and pulmonary morbidity.

Clinicians should consider CVID in patients with connective tissue diseases who present with recurrent infections, bronchiectasis, or hypogammaglobulinemia. Early diagnosis and timely initiation of immunoglobulin replacement therapy are essential to reduce infectious burden, limit pulmonary complications, and improve long-term outcomes.
